# The Association Between Child Maltreatment and Loneliness Across the Lifespan: A Systematic Review and Multilevel Meta-Analysis

**DOI:** 10.1177/10775595221103420

**Published:** 2022-06-02

**Authors:** Coriena de Heer, Shanshan Bi, Catrin Finkenauer, Lenneke Alink, Marlies Maes

**Affiliations:** 1 120705 Utrecht University, the Netherlands; 2 Leiden University, the Netherlands; 3 KU Leuven, Belgium

**Keywords:** child maltreatment, abuse, neglect, loneliness, lifespan, meta-analysis

## Abstract

While there is evidence that child maltreatment is positively associated with loneliness, the strength of this association is not yet clear. It is also unclear whether the magnitude and statistical significance of this association varies across groups of individuals. Therefore, this meta-analysis examines whether there are differences in loneliness between individuals with and without maltreatment histories, and which factors may influence the association between child maltreatment and loneliness. A three-level meta-analysis was conducted on 52 studies reporting 116 effect sizes (*N* = 1,705,493; *M*_*age*_ = 30.93; 49.6% females). Results showed a medium overall effect (*g* = 0.45, *p* < .001, 95% CI [0.36, 0.53]), indicating that individuals with maltreatment histories, on average, feel lonelier than individuals without maltreatment histories. Moderator analyses showed that effect sizes were larger for emotional abuse and emotional neglect as compared to other types of child maltreatment and decreased when participants were older at the time of loneliness assessment. These findings suggest that individuals with maltreatment histories, especially those who have been emotionally abused and/or emotionally neglected, are vulnerable to experiencing loneliness across the lifespan. The results also suggest that feelings of loneliness warrant attention in prevention and intervention programs for individuals with maltreatment histories.

Child maltreatment refers to all forms of abuse and neglect that cause actual or potential harm to those under the age of 18 years (World Health Organization, 2020). Meta-analyses of the worldwide prevalence of child maltreatment have reported mean prevalence rates ranging from 11.8% for sexual abuse to 26.7% for emotional abuse (Stoltenborgh et al., 2011, 2012). Research has indicated that exposure to child maltreatment is associated with more loneliness (Brown et al., 2016; Flett et al., 2016). Loneliness is the unpleasant feeling people experience when they perceive a quantitative or qualitative deficiency in their network of social relationships (Perlman & Peplau, 1981). This means that individuals have fewer relationships than they desire, or that they desire more intimacy than they experience in existing relationships.

Although there is a large body of research on the association between child maltreatment and loneliness, the strength of this association is not yet clear. It also remains unclear whether the positive association between child maltreatment and loneliness is universal, or whether it varies across, for example, type of child maltreatment, gender, and age of the individual. The lack of a systematic overview is worrying. Loneliness has considerable negative effects on individuals’ physical and mental health throughout the life course (for reviews, see Hawkley & Capitanio, 2015; Holt-Lunstad et al., 2015; Leigh-Hunt et al., 2017). If loneliness is not addressed in intervention programs for individuals with maltreatment histories who are at risk of loneliness, these individuals may remain vulnerable to develop depressive and anxiety symptoms, have a higher risk of cardiovascular diseases, and have an increased likelihood of early mortality. In this meta-analysis, we therefore quantify the association between child maltreatment and loneliness and identify groups of individuals with maltreatment histories that may be at increased risk of loneliness.

## Association Between Child Maltreatment and Loneliness

Theoretical perspectives provide a rationale for why individuals with maltreatment histories are likely to be at increased risk of experiencing loneliness. These perspectives are often linked to attachment theory, as most child maltreatment occurs by parents and the youngest children are the most vulnerable to maltreatment (U.S. Department of Health & Human Services, 2021). According to attachment theory, children build internal working models of relationships with others based on their relationship with their primary caregiver (Ainsworth & Bowlby, 1991). Maltreatment in childhood may undermine the quality of the relationship between children and their caregivers and yield an insecure and disorganized attachment relationship (Baer & Martinez, 2006; Cyr et al., 2010). Children with maltreatment histories, who experienced their caregivers’ neglectful or unpredictable abusive behavior, are more likely to develop expectations of others as unavailable, untrustworthy, and threatening (Capaldo & Perrella, 2018). In addition, caregivers who maltreat often fail to encourage and validate emotional expressions and to teach the child appropriate emotional regulation strategies (Dvir et al., 2014). Compared to children without maltreatment histories, children with maltreatment histories exhibit more emotion dysregulation and poorer emotion understanding (Dvir et al., 2014; Lavi et al., 2019). Both negative internal working models of others and deficits in emotion regulation and understanding could hinder the development and maintenance of future supportive relationships, such as with peers (Berlin et al., 1995; Capaldo & Perrella, 2018; Hay et al., 2004). This could make it difficult for individuals with maltreatment histories to fulfill their need for social relationships and put them at greater risk of experiencing loneliness.

Another perspective that could explain the positive association between child maltreatment and loneliness is the theory of latent vulnerability (McCrory & Viding, 2015). In this theory, latent vulnerability factors are considered processes that are initially adaptive in the maltreatment situation but later become a risk of problematic development. One of these processes is threat responsivity. Individuals with maltreatment histories are prone to hypervigilance to threat. This initially helps them to respond to unpredictable threatening behavior by their parents, but their tendency to overattribute threat may lead to more reactive aggression later in life (Richey et al., 2016). Reactive aggression may negatively influence existing and future social relationships (Fite et al., 2012), which makes it more likely that individuals with maltreatment histories perceive a deficit in their network of social relationships and feel lonely.

## Moderators of the Association Between Child Maltreatment and Loneliness

Several factors may influence the association between child maltreatment and loneliness across the lifespan. A set of potential moderators was investigated, which is described below. The moderators include child maltreatment characteristics (i.e., types of child maltreatment, age of onset, perpetrator, severity, frequency, and chronicity), loneliness characteristics (i.e., main types and relationship-specific types of loneliness), sample characteristics (i.e., gender, age, socioeconomic status, ethnic background, and clinical status), and study characteristics (i.e., measure of child maltreatment, reporter, study design, reliability of the measures, country, and year of publication). All moderators were selected a priori based on the current literature and preregistered on the Open Science Framework (https://osf.io/knq47).

### Child maltreatment characteristics

Child maltreatment characteristics, such as type, age of onset, perpetrator, severity, frequency, and chronicity, could impact the association between child maltreatment and loneliness. Typically, the literature distinguishes four main types of child maltreatment: emotional abuse, physical abuse, sexual abuse, and neglect. Emotional abuse is defined as the persistent refusal to consider a child’s basic emotional needs, for example, by intimidating or belittling a child (Cyr et al., 2010). Physical abuse is defined as any nonaccidental physical injury inflicted by an adult on a child, for example, striking or biting a child. Sexual abuse is defined as the involvement of a child in any sexual behavior aimed to satisfy the adult’s sexual needs or to make financial profit (Abbasi et al., 2015). Neglect is defined as the failure of a parent or other person with responsibility for the child to meet the child’s basic needs, including food, clothing, shelter, medical care, and supervision, by which the child’s health, safety, and well-being are threatened with harm. Neglect includes both emotional neglect (i.e., failure to meet children’s emotional needs, such as affection) and physical neglect (i.e., failure to meet children’s physical needs, such as adequate nutrition).

All four types have been shown to be positively related to loneliness (Brown et al., 2016; Hanlon et al., 2020). Previous research suggests that emotional abuse and emotional neglect are stronger predictors of insecure attachment relationships than physical abuse, sexual abuse, and physical neglect (Lowell et al., 2014). In addition, meta-analyses have indicated that, compared to the other types of child maltreatment, emotional abuse and emotional neglect are more strongly related to internalizing problems, such as depression and anxiety (Gardner et al., 2019; Humphreys et al., 2020). Therefore, we hypothesized that the effect of having experienced child maltreatment on loneliness would be the strongest for individuals who have been emotionally abused or emotionally neglected. Also, the age of onset may influence the association between child maltreatment and loneliness. The earlier the maltreatment occurs, the more likely it hinders the acquisition of skills that are important in developing and maintaining social relationships, such as emotion regulation, which may make children more vulnerable to experiencing loneliness (Bolger et al., 1998; Kaplow & Widom, 2007).

Furthermore, research has indicated that child maltreatment perpetrated by someone who is emotionally close to the child, such as a caregiver, is related to worse outcomes compared to child maltreatment perpetrated by a more emotionally distant person (Edwards et al., 2012; Goldsmith et al., 2012; Ullman, 2007). It is theorized that maltreatment by a caregiver puts children in a position in which they are dependent upon the caregiver for survival, and therefore, they have to maintain the relationship with the caregiver (Gagnon et al., 2017). This requires children to minimize consciousness of the maltreatment and use processes such as dissociation and alexithymia (i.e., difficulties to identify and describe emotions) to inhibit awareness (Freyd et al., 2001; Goldsmith et al., 2012). Such reactions could interfere with the formation of future relationships making them vulnerable to experiencing loneliness. Thus, stronger effects of having experienced child maltreatment on loneliness were expected for individuals who have been maltreated by their caregivers than for individuals who have been maltreated by noncaregivers.

In addition, research has shown that, in general, more severe, more frequent, and more chronic experiences of child maltreatment are related to more severe outcomes (Bryant & Range, 1997; English et al., 2005; Éthier et al., 2004). For example, more chronic experiences of child maltreatment predicted more disturbed peer relations that increased the risk of feelings of loneliness (Graham et al., 2010). Therefore, we hypothesized that the effect of having experienced child maltreatment on loneliness would be the strongest for individuals who have experienced more severe, more frequent, and/or more chronic child maltreatment.

### Loneliness characteristics

Different types of loneliness can be distinguished, and the association between child maltreatment and loneliness may differ per type. The two main types of loneliness are emotional and social loneliness (Maes, Qualter, et al., 2019). Emotional loneliness refers to the feeling of lacking a close relationship in which emotional support, affection, and intimacy are provided. This perceived absence of someone who really knows and understands you can refer to different relationships across the lifespan, for example, a parent, best friend, or romantic partner. Social loneliness refers to the feeling of lacking a network of social relationships, so lacking the feeling of belongingness or people with whom one can spend time, for example, a family or peers. Individuals with maltreatment histories often experience the maltreatment as a betrayal of trust from their caregivers, and they may generalize this distrust to other people (Baugh et al., 2019; Gobin & Freyd, 2014). It could be argued that lower trust in others interrupts close relationships in particular, and therefore child maltreatment may be more strongly related to emotional loneliness than to social loneliness. However, research examining the suggestion is lacking.

In addition to these main types, relationship-specific types of loneliness can be distinguished as loneliness can be experienced in different relationship contexts (i.e., with peers, family, or a romantic partner). Child maltreatment is associated with disrupted peer and romantic relationships (Bolger & Patterson, 2001; Riggs, 2010). This could make it harder for individuals with maltreatment histories to fulfill their needs concerning these relationships and put them at greater risk of experiencing loneliness within peer and romantic relationships. However, it could be argued that child maltreatment has the most impact on family relationships. Most of the child maltreatment occurs within families (Miller-Perrin & Perrin, 2013). Therefore, it is possible that child maltreatment interrupts individuals’ need for close family relationships in particular, and that individuals with maltreatment histories are most vulnerable to experience loneliness within family relationships.

### Sample characteristics

The literature suggests that several sample characteristics could impact the association between child maltreatment and loneliness, namely gender, age, socioeconomic status, ethnic background, and clinical status. These moderators were mainly examined in an explorative way.

Child maltreatment seems to have a stronger association with girls’ compared to boys’ internalizing problems (Hagborg et al., 2017; Maschi et al., 2008). Additionally, research suggests that girls value interpersonal relationships more than boys (Cross & Madson, 1997). Considering child maltreatment negatively interferes with interpersonal relationships (Alto et al., 2018), girls with maltreatment histories may be more vulnerable to experience loneliness relative to boys with maltreatment histories. Furthermore, the age of the participants at the time of loneliness assessment may influence the association between child maltreatment and loneliness. During childhood, relationships with parents are most important (Collins & Laursen, 2004). At the same time, child maltreatment most often occurs by parents (U.S. Department of Health & Human Services, 2021). The central role parents play in children’s life may make children with maltreatment histories especially vulnerable to experiencing loneliness. When children grow older and turn into adolescents and adults, their social networks expand beyond their parents with friends and/or a romantic partner. Those additional types of relationships might buffer against feelings of loneliness, potentially making the association between child maltreatment and loneliness less strong with age.

In addition to gender and age of the participants, the socioeconomic status of the participants may impact the association between child maltreatment and loneliness. Often in research, the socioeconomic status of children and their families at the time of child maltreatment is unknown or not reported. Nevertheless, socioeconomic status at the time the study was conducted often is reported. Because childhood socioeconomic status is related to adulthood socioeconomic status (Elovainio et al., 2012), socioeconomic status later in life may be considered as a proxy for socioeconomic status at the time of the child maltreatment. Low socioeconomic status is, like child maltreatment, a risk factor for mental health problems (Kivimäki et al., 2020). Thus, participants with maltreatment histories from lower socioeconomic status families are likely to face multiple risk factors at the same time. Accumulation of risk factors is related to more deleterious outcomes later in life (Appleyard et al., 2005). Therefore, we hypothesized that the association between child maltreatment and loneliness may be stronger for participants with lower socioeconomic status than for participants with higher socioeconomic status.

Furthermore, research suggests that the impact of child maltreatment may differ according to ethnic background. Of the men who experienced childhood sexual abuse, White men more frequently mentioned loneliness than Black and Latino men (Payne et al., 2014). White children with histories of neglect also showed in general more mental health consequences although Black children with histories of neglect showed an increased risk of generalized anxiety and dysthymia (Widom et al., 2013). Because the direction of the moderator ethnic background seems to be unclear, we will investigate the moderating role of ethnic background exploratively. Finally, individuals with maltreatment histories who additionally have a clinical disorder, such as depression, may find it especially difficult to develop and maintain relationships. They may, therefore, be particularly vulnerable to experiencing loneliness. Thus, child maltreatment may be more strongly related to loneliness in clinical samples than in non-clinical samples. However, research on this topic is lacking.

### Study characteristics

In addition to sample characteristics, several study characteristics could influence the association between child maltreatment and loneliness, including measure of child maltreatment, reporter, study design, reliability of the measures, country in which the study was conducted, and year of publication.

Growing evidence suggests that the type of measure of child maltreatment may moderate the association between child maltreatment and adult mental health (Latham et al., 2020; Newbury et al., 2018; Reuben et al., 2016). Retrospective measures of child maltreatment seem to be more strongly associated with poorer adult mental health and more adult psychopathology than prospective measures of child maltreatment. Possibly, the way maltreatment was experienced and memorized may impact later mental health (Danese & Widom, 2020). Therefore, we expected a stronger association between child maltreatment and loneliness for studies that used a retrospective measure than for studies that used a prospective measure of child maltreatment.

In addition, whether or not the reporter of both constructs is the same may impact the association between child maltreatment and loneliness. Due to common method variance, the association between child maltreatment and loneliness may be stronger when both constructs were measured using self-reports than when one of the constructs was measured using other-reports, for example, official records. Also, studies with a cross-sectional study design collected data on child maltreatment and loneliness at the same time point causing possible overlap in the systematic error which inflates the strength of the association (Lindell & Whitney, 2001). Therefore, child maltreatment may be more strongly associated with loneliness in cross-sectional than in longitudinal studies.

Furthermore, the reliability of the used measures may impact the association between child maltreatment and loneliness because lower reliability (i.e., more measurement error) reduces the effect size of an association (Hunter & Schmidt, 2004). Also, the association between child maltreatment and loneliness may differ across countries. Because, for example, different behaviors may be considered as more acceptable across countries (Fakunmoju et al., 2013) and might therefore have a different impact. Research on cross-country differences in the association between child maltreatment and loneliness, however, is lacking. Finally, the year of publication may moderate the association between child maltreatment and loneliness. Earlier published studies often relied on weaker research methods which could inflate effect sizes (Ioannidis, 2005).

## Present Study

This meta-analysis aimed to investigate whether there are differences in loneliness between individuals with and without maltreatment histories and to gain insight into the overall size of this effect. While there is evidence for a positive association between child maltreatment and loneliness, a quantification of this association is lacking. In addition to investigating the overall effect of having experienced child maltreatment on loneliness, we examined several child maltreatment, loneliness, sample, and study characteristics that could influence this association.

## Method

This study followed the Preferred Reporting Items for Systematic Reviews and Meta-Analyses (PRISMA) guidelines (Page et al., 2021). We preregistered the study on the Open Science Framework (https://osf.io/knq47).

### Literature Search

We conducted searches in Cochrane Central Register of Controlled Trials, EMBASE, ERIC, MEDLINE, PsycINFO, PubMed, and Web of Science on 2 November 2020. Search terms were a combination of keywords regarding loneliness (lonel* or “perceived social isolation”), and child maltreatment (maltreat* or mistreat* or abus* or neglect* or abandoned or incest* or rape* or “shaken baby syndrome”) and (child* or kid* or teen* or adolescen* or youth). Full search strings for each database can be found in Appendix A. We also screened the reference lists of prior meta-analyses and systematic reviews of child maltreatment and loneliness for additional potentially relevant articles.

### Selection of Articles

Articles were eligible for this meta-analysis if they met the following inclusion criteria: the article (a) was published in English; (b) was published in a peer-reviewed journal; (c) reported quantitative research; (d) included a measurement of loneliness; (e) included a measurement of child maltreatment; and (f) included both participants with and without maltreatment histories. Articles on trauma, adverse childhood experiences, or milder types of behavior reflecting child maltreatment (e.g., harsh parenting and psychological control) were excluded if child maltreatment was not explicitly mentioned in the article. In addition, we excluded articles that only measured witnessing of domestic violence or intimate partner violence because this meta-analysis focused more generally on emotional abuse, physical abuse, sexual abuse, and neglect.

**Figure 1.**
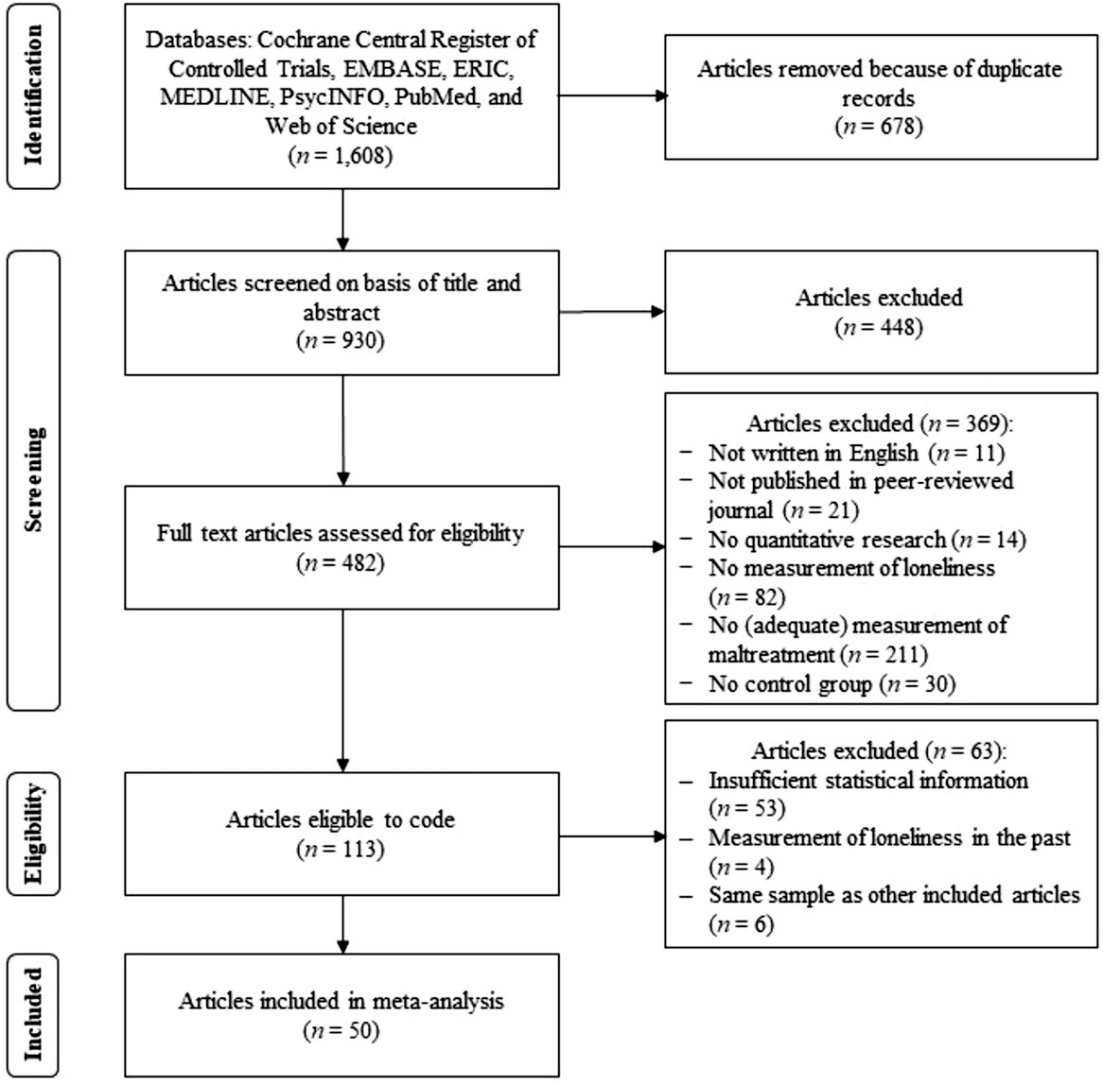
PRISMA Flow Diagram. *Note. n* = number of articles.

The data search yielded 930 nonduplicate hits (for the flow diagram, see Figure 1). Two of the authors (C. H., S. B.) independently conducted the first selection in which they screened the title and the abstract and the second selection in which they screened the full text for eligibility. Disagreements were resolved by consensus. In the first selection, only the first three inclusion criteria were assessed, and 482 articles met these criteria (κ = .79). In the second selection, 369 articles were excluded (κ = .80), mainly because no (adequate) measurement of maltreatment was available. Thus, a total of 113 articles were eligible for inclusion in the current meta-analysis. Eighty-six articles contained insufficient information to calculate the effect size. We requested data via email from the authors, and the authors of 33 articles provided the requested data. Four articles were excluded because they measured loneliness in the past and were therefore not comparable to the other articles that measured current loneliness. Finally, we excluded six articles because the same sample of participants was used in multiple articles, and the other article had more complete data and/or a larger sample size. When articles contained the same amount of data and the samples were equally large, the first published article was included. The final data set consisted of 50 articles and reported 116 effect sizes (*k*) from 52 studies (*n*).

### Coding

All articles were coded by the first author. A coding manual and scheme were developed, and a second coder (S. B.) independently double coded 25% of the articles to calculate inter-rater reliability. For categorical variables, Cohen’s Kappa indicated substantial to perfect agreement (κ = .63 to 1.00), except for measure of child maltreatment (κ = .28) for which we adjusted the coding manual to avoid ambiguity for further coding. For continuous variables, intra-class correlations indicated excellent reliability (ICC = .95 to 1.00). Disagreements were discussed and resolved among the authors. Data were extracted for child maltreatment characteristics (e.g., type of child maltreatment, perpetrator), loneliness characteristics (type of loneliness, relationship in which loneliness was experienced), sample characteristics (e.g., gender, age), and study characteristics (e.g., study design, year of publication). A concise version of the coding manual is available in Appendix B.

### Effect Size Calculations

Hedges’ *g*, which is a small-sample correction of Cohen’s *d* (Hedges & Olkin, 1985), was used as effect size in the current meta-analysis. To calculate this effect size, we subtracted the loneliness mean of individuals without maltreatment histories from that of individuals with maltreatment histories and divided the result by the pooled weighted standard deviation. Thus, a positive effect size indicates a higher loneliness mean for individuals with maltreatment histories than individuals without maltreatment histories. For all effect sizes, we applied Hedges’ small-sample correction. The effect sizes were weighted by the inverse variance to ensure that samples with more precise estimates got a greater weight in the analyses (Marín-Martínez & Sánchez-Meca, 2010). We considered an effect size of *g =* 0*.*2 as small, of *g =* 0*.*5 as medium, and of *g =* 0*.*8 as large (Tomczak & Tomczak, 2014). Different statistics were used to calculate Hedges’ *g* and the corresponding standard error. The majority of studies provided means, standard deviations, and sample sizes for individuals with and without maltreatment histories separately or other effect sizes, such as correlations and odds ratios.

### Statistical Analyses

Most studies reported multiple effect sizes, and therefore these effect sizes were not independent as they were based on the same sampling and data collection approach. Consequently, we conducted a multilevel meta-analysis, an approach that accounts for the dependency between effect sizes (Van den Noortgate et al., 2013). In this study, we used a three-level meta-analysis that considers three different variance components: Sampling variance of the effect sizes, variance between effect sizes of the same study, and variance between studies (Assink & Wibbelink, 2016).

To investigate differences in loneliness between individuals with and without maltreatment histories and to investigate moderator effects, we performed several analyses (Assink & Wibbelink, 2016). First, we calculated the overall effect of having experienced child maltreatment on loneliness. Second, we tested the significance of the within-study variance and the between-study variance by conducting two log-likelihood-ratio tests, and we examined how the total variance was distributed over the three different variance components. The sampling variance is no single value because it depends on the sample size. To make an estimate of the sampling variance, we used the median sampling variance. Third, if there was heterogeneity of within-study and/or between-study variance, we conducted moderator analyses. To avoid multicollinearity, we conducted univariate moderator analyses by including the moderators one by one as predictors in the model. Regarding the moderators, at least five effect sizes were required for a particular category to be included in the analyses (Maes, Nelemans, et al., 2019). Analyses were conducted in R version 4.0.3 with the metafor package using restricted maximum likelihood as estimation method (Assink & Wibbelink, 2016; Viechtbauer, 2010).

Furthermore, we examined whether there were outliers, that is, effect sizes deviating three or more standard deviations from the mean. For the main analyses, results of both analyses with and without outliers are reported, and for the moderator analyses, only results of the analyses without outliers are reported. To assess publication bias, we created a funnel plot and conducted an extension of Egger’s regression test using effect size as a predictor. In the funnel plot, the individual effect sizes were plotted with effect size (*g*) on the horizontal axis and the standard error on the vertical axis. If no publication bias is present, the funnel plot is shaped like a funnel. An extension of Egger’s regression test was conducted to test statistically whether the effect sizes were asymmetrically distributed. On the Open Science Framework, both the dataset (https://osf.io/mqdsw/) and the analysis script (https://osf.io/6gvqe/) are available.

## Results

### Descriptives

Study and sample characteristics for each included study can be found in Appendix C. Sample size ranged from 45 to 1,338,785 (median *N* = 519), with a total of 1,705,493 participants included in the present meta-analysis. Participants’ mean age, as reported in 45 studies, was 30.93 years (*SD* = 19.86), and 49.6% of the participants were female. The studies were published between 1992 and 2020. Most studies were conducted in Europe (*n* = 20) and North America (*n* = 18), followed by studies conducted in Asia (*n =* 11) and South America (*n* = 3).

### Association Between Child Maltreatment and Loneliness

The analysis based on the standardized mean differences from 52 studies yielded a significant medium effect, *g* = 0.49 (*SE* = 0.06, *p* < .001, 95% CI [0.37, 0.62]), demonstrating that individuals with maltreatment histories, on average, feel lonelier than individuals without maltreatment histories. Two effect sizes were considered as outliers as they were more than three standard deviations above the mean effect size. Analyzing the data without these two outliers again yielded a significant medium effect, *g* = 0.45 (*SE* = 0.04, *p* < .001, 95% CI [0.36, 0.53]). For further analyses, we excluded the outliers. Looking at the variances at the different levels, we found significant variance within studies, estimate = 0.01, χ^2^(1) = 53.92, *p* < .001, and between studies, estimate = 0.08, χ^2^(1) = 42.05, *p* < .001. The sampling variance represented 15.85%, the within-study variance 8.92%, and the between-study variance 75.23% of the total variance. Thus, on top of the sampling variance, there was variance because of variability between effect sizes within studies and between studies. Moderator analyses were conducted to try to explain the within- and between-study variance.

### Moderators of the Association Between Child Maltreatment and Loneliness

For most of the child maltreatment characteristics (i.e., for perpetrator, age of onset, severity, frequency, and chronicity), insufficient data were available to include them as a moderator. The relationship in which loneliness was experienced and the reporter of loneliness were also excluded in the moderator analyses because insufficient effect sizes were available. Because sufficient data were available to include mean age as a moderator, we deviated from the preregistration and did not include age categories. Age categories only served as a backup in case insufficient data were available for mean age. We performed univariate moderator analyses for the other moderators (for the results, see Table 1). Most moderators were not significantly related to the difference in loneliness between individuals with and without maltreatment histories, namely type of loneliness, gender, socioeconomic status, ethnic background, clinical status, reliability of the measures, country, and year of publication. Significant moderators were type of child maltreatment, age of the participants, measure of child maltreatment, reporter of child maltreatment, and study design.

**Table 1.** Separate Regression Analyses for the Moderators Predicting Differences in Loneliness Between Individuals With and Without Maltreatment Histories.

Moderator	*k*	*b*	*SE*	95% CI	*F*	*df*	*p*
Maltreatment type	96				5.43	4, 91	.001
Emotional abuse	19	0.54_a_	0.05	[0.43, 0.65]			<.001
Physical abuse	24	0.39_b_	0.05	[0.29, 0.49]			<.001
Sexual abuse	34	0.35_b_	0.05	[0.26, 0.45]			<.001
Emotional neglect	12	0.54_a_	0.06	[0.42, 0.67]			<.001
Physical neglect	7	0.37_b_	0.08	[0.21, 0.52]			<.001
Loneliness type	100				0.31	2, 97	.735
Emotional	8	0.39	0.10	[0.19, 0.58]			<.001
Social	30	0.45	0.07	[0.32, 0.58]			<.001
General	62	0.45	0.06	[0.33, 0.57]			<.001
Gender	114	0.19	0.18	[-0.15, 0.54]	1.21	1, 112	.274
Age	105	−0.01	0.00	[-0.01, −0.00]	4.19	1, 103	.043
Socioeconomic status	63				1.13	2, 60	.330
>75% low	9	0.22	0.13	[-0.04, 0.48]			.090
>75% middle/high	15	0.47	0.11	[0.26, 0.68]			<.001
Mixed	39	0.41	0.07	[0.28, 0.54]			<.001
Ethnic background	57				0.39	1, 55	.533
≤75% majority	23	0.39	0.08	[0.24, 0.55]			<.001
>75% majority	34	0.46	0.06	[0.33, 0.59]			<.001
Clinical status	114				2.95	2, 111	.057
Nonclinical	94	0.48	0.04	[0.39, 0.57]			<.001
Clinical	13	0.31	0.15	[0.01, 0.61]			.043
Mixed	7	0.01	0.20	[-0.40, 0.41]			.963
Maltreatment measure	113				7.41	1, 111	.008
Retrospective	101	0.48	0.04	[0.39, 0.57]			<.001
Prospective	12	0.15	0.11	[-0.08, 0.37]			.191
Maltreatment reporter	108				6.56	1, 106	.012
Self-report	99	0.48	0.05	[0.39, 0.57]			<.001
Records	9	0.13	0.13	[-0.12, 0.39]			.306
Study design	114				6.25	1, 112	.014
Cross-sectional	86	0.50	0.05	[0.41, 0.59]			<.001
Longitudinal	28	0.24	0.09	[0.05, 0.42]			.012
Reliability loneliness	68	0.25	0.34	[-0.43, 0.92]	0.53	1, 66	.467
Reliability maltreatment	26	−0.02	0.35	[-0.74, 0.69]	0.01	1, 24	.943
Country	114				0.32	2, 111	.725
Europe	47	0.42	0.07	[0.28, 0.57]			<.001
North America	38	0.42	0.08	[0.27, 0.58]			<.001
Asia and South America	29	0.50	0.08	[0.34, 0.67]			<.001
Publication year	114	−0.00	0.01	[-0.01, 0.01]	0.00	1, 112	.957

*Note. k* = number of effect sizes; *b* = regression coefficient (can be interpreted as the mean effect size for each category for the categorical variables); CI = confidence interval. Effects sizes are significantly different if they do not have the same subscript.

First, the difference between loneliness experienced by individuals with maltreatment histories compared to individuals without maltreatment histories was greater for emotional abuse and emotional neglect than for physical abuse, sexual abuse, and physical neglect. Second, the significant moderator age indicated that the difference in loneliness between individuals with and without maltreatment histories became smaller when participants were older at the time of loneliness assessment. Third, studies that measured child maltreatment retrospectively found that individuals with maltreatment histories were lonelier than individuals without maltreatment histories, while studies that measured child maltreatment prospectively did not find a difference in loneliness between individuals with and without maltreatment histories. Fourth, studies that used self-reports to measure child maltreatment found that individuals with maltreatment histories were lonelier than individuals without maltreatment histories, while studies that used official records to measure child maltreatment did not find a difference in loneliness between individuals with and without maltreatment histories. Fifth, cross-sectional studies reported a stronger association between child maltreatment and loneliness than longitudinal studies.

### Publication Bias

**Figure 2.**
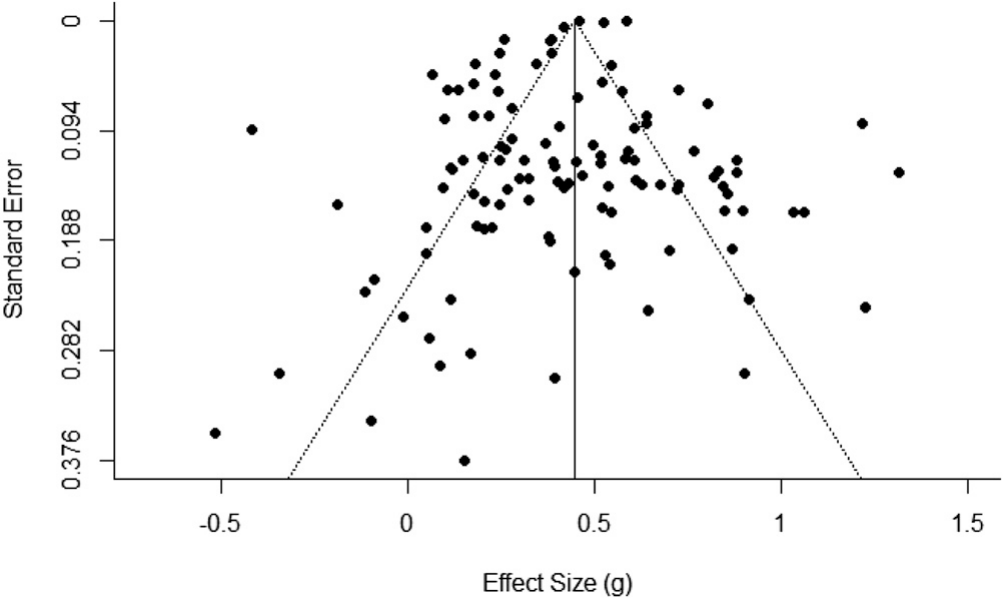
Funnel Plot of the Effect Sizes Without Outliers.

Studies appeared to be symmetrically distributed around the combined effect size (see Figure 2). Therefore, no publication bias was suggested. In addition, the extension of Egger’s regression test with standard error added as moderator was nonsignificant, *F*(1, 112) = 0.45, *p* = .502. This also suggested that there was no publication bias. Thus, the data showed little evidence for the presence of publication bias in the current meta-analysis.

## Discussion

The present meta-analysis synthesized the research on loneliness in individuals with maltreatment histories compared to individuals without maltreatment histories. Overall, we found a medium significant effect indicating that individuals with maltreatment histories, on average, experience more loneliness than individuals without maltreatment histories. Differences in loneliness between individuals with and without maltreatment histories varied as a function of the type of child maltreatment, the age of the participants, the measure of child maltreatment, the reporter of child maltreatment, and the study design. No significant effects were found for the other moderators, that is, type of loneliness, gender, socioeconomic status, ethnic background, clinical status, reliability of the measures, country, and year of publication.

### Association Between Child Maltreatment and Loneliness

We found a positive association between child maltreatment and loneliness demonstrating that individuals with maltreatment histories are more vulnerable to experiencing loneliness than individuals without maltreatment histories. Previous research suggests several factors that could explain why individuals with maltreatment histories are at risk of feelings of loneliness. Child maltreatment may negatively impact the quality of parent-child relationships that could lead to the development of negative expectations of other people (Capaldo & Perrella, 2018). Individuals with maltreatment histories also show deficits in their emotion regulation and emotion understanding (Dvir et al., 2014; Lavi et al., 2019). In addition, individuals with maltreatment histories are more likely to overattribute threat and show more reactive aggression (Richey et al., 2016). All these factors may hinder the development and maintenance of (intimacy in) social relationships and make it more likely that individuals with maltreatment histories perceive a deficit in their network of social relationships and experience loneliness.

Most research considers child maltreatment as a risk factor for loneliness. However, based on our study we can only conclude that child maltreatment and loneliness are associated because we did not investigate directionality. It is also possible that loneliness increases the likelihood for individuals to experience child maltreatment. According to the ecological model of child maltreatment, children’s characteristics can increase the risk of maltreatment (Belsky, 1980). Research supports this model by demonstrating that child factors contribute to child maltreatment (Pittner et al., 2020). For example, children’s internalizing problems may cause higher levels of parenting stress, which are related to an increased child maltreatment potential (Miragoli et al., 2016). Loneliness could be considered as an internalizing problem (Blossom & Apsche, 2013), and children who feel lonely may be more at risk of child maltreatment. It is also possible that child maltreatment and loneliness mutually influence each other over time as predicted by the transactional model (Sameroff, 1975).

### Moderators of the Association Between Child Maltreatment and Loneliness

In addition to investigating the overall effect of having experienced child maltreatment on loneliness, we investigated several child maltreatment, loneliness, sample, and study characteristics that may moderate this effect.

First, we found that individuals who have experienced child maltreatment regardless of the child maltreatment type experienced more loneliness than individuals who have not experienced child maltreatment. However, consistent with our hypothesis, individuals who have been emotionally abused and/or emotionally neglected were more vulnerable to experiencing loneliness compared to individuals who have been physically abused, sexually abused, and/or physically neglected. Previous research suggests that emotional abuse and emotional neglect, in particular, may hinder secure attachment relationships between children and their parents (Lowell et al., 2014), undermine emotion regulation and emotion understanding (Heim et al., 2013), and yield negative beliefs about yourself as unworthy and incompetent and other people (Wright et al., 2009). Insecure attachment, deficits in emotional skills, and negative beliefs about yourself and others may interfere with the formation and maintenance of future relationships and are all related to an increased risk of experiencing loneliness (Akdoğan, 2017; Qualter et al., 2009; Wols et al., 2015).

Second, our results indicated that differences in loneliness between individuals with and without maltreatment histories did not vary according to the type of loneliness (i.e., emotional and social loneliness). Thus, the findings do not support our hypothesis that individuals with maltreatment histories experience more emotional loneliness than social loneliness because child maltreatment may influence trust in other people which could interfere with maintaining close relationships (Gobin & Freyd, 2014). Possibly the lack of trust in other people not only hinders the development of close relationships but impacts the development of social relationships in general, and therefore, is related to both types of loneliness. Thus, child maltreatment is associated both with the lack of close relationships and the lack of a network of social relationships.

Third, gender, socioeconomic status, ethnic background, and clinical status did not moderate the association between child maltreatment and loneliness. Thus, our results suggest that the vulnerability to experiencing loneliness for individuals with maltreatment histories compared to individuals without maltreatment histories is comparable across a wide range of individual characteristics. The only sample characteristic that emerged as a significant moderator was the age of the participants at the time of loneliness assessment. The difference in loneliness between individuals with and without maltreatment histories became smaller when participants were older. However, age has a very small effect size and even at an old age (i.e., above 65 years of age), individuals with maltreatment histories were lonelier than individuals without maltreatment histories. Another meta-analysis found that individuals with maltreatment histories showed poorer performance in social understanding than individuals without maltreatment histories but particularly children compared to adolescents and adults (Luke & Banerjee, 2013). A deficit in social understanding skills may be a risk factor of experiencing loneliness (Lim et al., 2020). This deficit may be particularly pronounced in childhood and could hinder the development of social relationships that may increase feelings of loneliness. Another possible explanation could be that the older participants are, the more likely it is that they have received some intervention to process the trauma that also addressed factors that increase the risk of loneliness, for example, negative beliefs about the self and others.

Fourth, several of the study characteristics were not significant moderators, namely the reliability of the loneliness and child maltreatment measures, the country in which the study was conducted, and the year of publication. The measure of child maltreatment, reporter of child maltreatment, and study design were significant moderators. Studies that measured child maltreatment retrospectively, studies that used self-reports to measure child maltreatment, and/or cross-sectional studies reported a stronger association between child maltreatment and loneliness than studies that measured child maltreatment prospectively, studies that used records to measure child maltreatment, and/or longitudinal studies. The moderators measure of child maltreatment, reporter of child maltreatment, and study design very often coincided. Most cross-sectional studies used retrospective self-reports to measure child maltreatment, while most longitudinal studies used prospective informant-reports to measure child maltreatment. Because the moderators strongly overlapped, it is unknown which of these moderators mainly drove the difference in loneliness between individuals with and without maltreatment histories, and therefore these findings should be interpreted with caution.

### Limitations and Future Directions

Several limitations of the present meta-analysis should be noted. First, most of the included studies were cross-sectional, and therefore we only know there is an effect, but we cannot interpret the direction of the effect. The included longitudinal studies often did not provide loneliness scores at the time the child maltreatment was measured, and therefore we could not investigate causality. Although most research assumes that loneliness is a consequence of child maltreatment, longitudinal studies are necessary to establish the temporal and causal sequences.

Second, of the child maltreatment characteristics described in the Introduction, we only investigated the type of child maltreatment as a moderator. In the current meta-analysis, insufficient studies reported the age of onset, perpetrator, severity, frequency, and chronicity of child maltreatment. From previous research, we know that different characteristics of child maltreatment are often intertwined and may impact the strength of the association between child maltreatment and loneliness (English et al., 2005; Jackson et al., 2014). In addition, many individuals with maltreatment histories experience more than one type of child maltreatment (Jackson et al., 2014), and exposure to multiple types of child maltreatment is related to more adverse outcomes (Kisely et al., 2018). However, we could not investigate whether experiencing multiple types of child maltreatment was associated with a higher risk of experiencing loneliness because too little data was available. Research is needed that includes a wide range of child maltreatment characteristics to further illuminate the association between child maltreatment and loneliness and to investigate the role of the (interactions of the) different child maltreatment characteristics in this association.

Additionally, to further understand the association between child maltreatment and loneliness, we recommend exploring different aspects of loneliness as well. In the current meta-analysis, the first step has been made by distinguishing different types of loneliness, namely emotional and social loneliness. Another important aspect to explore is the severity of loneliness. The studies included in the present meta-analysis measured the severity of loneliness in terms of the frequency of the loneliness experience but did not capture other dimensions of the loneliness experience such as intensity or duration (Qualter et al., 2021). Intensity refers to the level of distress feelings of loneliness cause, and duration refers to the timespan feelings of loneliness last. Especially with regard to duration, increasing research emphasizes the importance to distinguish between transient loneliness and chronic loneliness. Transient loneliness refers to short and infrequent feelings of loneliness, while chronic loneliness refers to more stable feelings of loneliness for a longer period. Chronic loneliness, compared to transient loneliness, is related to worse health outcomes (Martín-María et al., 2020). However, no study in the current meta-analysis distinguished between transient and chronic loneliness. Further research should investigate which aspects of loneliness are most strongly associated with child maltreatment. This knowledge will be particularly important for increasing the effectiveness of prevention and intervention programs.

Third, although moderators such as country, socioeconomic status, and ethnic background were not significant, we must be cautious to generalize our findings to the global population. For example, nearly three-quarters of the included studies were conducted in Europe and North America. Especially Africa and South America were largely underrepresented since we only included a few studies conducted in South America and no studies conducted in Africa. Furthermore, about half of the studies did not provide information on the socioeconomic status or the ethnicity of the participants. Of the studies that did include information on the ethnic background of the participants, about half of the studies included samples in which more than 75% of the participants had an ethnic majority background. Thus, we would like to strongly recommend that researchers report on the demographics of the sample in their studies and include ethnic minority populations and populations in Africa and South America.

Lastly, future research is needed to investigate potential mechanisms that may explain the increased risk of loneliness for individuals with maltreatment histories. One mechanism that may explain why child maltreatment and loneliness are associated is the development of an insecure parent-child attachment (Capaldo & Perrella, 2018). Insecure parent-child attachment may impair the ability to develop and maintain social relationships and puts individuals with maltreatment histories at greater risk of experiencing loneliness. Furthermore, research has indicated that a negative self-concept, such as low self-esteem (Luo et al., 2020) and low sense of mattering (Flett et al., 2016), may mediate the association between emotional maltreatment and loneliness. Nevertheless, it still remains unclear whether a negative self-concept could also explain the association between the other types of child maltreatment and loneliness. In addition, research has shown that cognitive aspects, such as rumination and hopelessness, mediate the association between child maltreatment and symptoms of depression and anxiety (Hamilton et al., 2013; Kim et al., 2017). Future research should investigate whether these cognitive aspects also explain the increased risk of loneliness in individuals with maltreatment histories.

### Clinical Implications

The findings may have clinical implications for professionals working with individuals with maltreatment histories. This study indicates that these individuals are more vulnerable to experiencing loneliness than individuals without maltreatment histories. Loneliness could play an important role in the development of negative outcomes related to child maltreatment. Research showed that loneliness mediates the association between child maltreatment and internalizing and externalizing problems in childhood and mental and physical health problems in adulthood (Appleyard et al., 2010; Shevlin et al., 2015; Thoresen et al., 2018). Treating loneliness may be an effective strategy in preventing further potential negative consequences of child maltreatment. Therefore, clinical assessments should include asking individuals with maltreatment histories about their feelings of loneliness, and when necessary, addressing loneliness in interventions would be promising. Noteworthy, it may not be enough to only focus on encouraging and helping individuals with maltreatment histories build a social network and increase social contacts, as people still can feel lonely if they do not feel connected to the people in their social network or miss close relationships.

A promising evidence-based intervention to address loneliness in children and adolescents who have experienced child maltreatment is Trauma-Focused Cognitive Behavioral Therapy (TF-CBT; Cohen & Mannarino, 2017). In addition to helping children and adolescents gain social skills and seek social support, it also focuses on helping individuals with maltreatment histories to think more positively about themselves and others which seems to be the most effective aspect of an intervention to reduce loneliness (Masi et al., 2011).

### Conclusion

Individuals with maltreatment histories were found to be more vulnerable to experiencing loneliness compared to individuals without maltreatment histories, especially individuals who have been emotionally abused and/or emotionally neglected. An important next step is to study the underlying mechanisms of the association between child maltreatment and loneliness to deepen our understanding of why individuals with maltreatment histories are at an increased risk of experiencing loneliness. This knowledge is needed to inform researchers and professionals about how to prevent or address loneliness in prevention and intervention programs. In the meantime, professionals should be aware of the risk of feelings of loneliness in individuals with maltreatment histories.

## Supplemental Material

sj-pdf-1-cmx-10.1177_10775595221103420 – Supplemental Material for The Association Between Child Maltreatment and Loneliness Across the Lifespan: A Systematic Review and Multilevel Meta-Analysis Supplemental Material, sj-pdf-1-cmx-10.1177_10775595221103420 for The Association Between Child Maltreatment and Loneliness Across the Lifespan: A Systematic Review and Multilevel Meta-Analysis by Coriena de Heer, Shanshan Bi, Catrin Finkenauer, Lenneke Alink and Marlies Maes in Child Maltreatment

sj-pdf-2-cmx-10.1177_10775595221103420 – Supplemental Material for The Association Between Child Maltreatment and Loneliness Across the Lifespan: A Systematic Review and Multilevel Meta-Analysis Supplemental Material, sj-pdf-2-cmx-10.1177_10775595221103420 for The Association Between Child Maltreatment and Loneliness Across the Lifespan: A Systematic Review and Multilevel Meta-Analysis by Coriena de Heer, Shanshan Bi, Catrin Finkenauer, Lenneke Alink and Marlies Maes in Child Maltreatment
