# Supplementary material for: The Association Between Child Maltreatment and Loneliness Across the Lifespan: A Systematic Review and Multilevel Meta-Analysis
Source: Child Maltreat. 2022 Jun 2;29(2):388–404. doi: 10.1177/10775595221103420 (PMC11539460; doi:10.1177/10775595221103420)
Supplement: sj-pdf-1-cmx-10.1177_10775595221103420 – Supplemental Material for The Association Between Child Maltreatment and Loneliness Across the Lifespan: A Systematic Review and Multilevel Meta-Analysis [file sj-pdf-1-cmx-10.1177_10775595221103420.pdf]

Supplementary File A. Preregistration

Preregistration Template from AsPredicted.org

1) ***Data collection. Have any data been collected for this study already? Note: 'Yes' is a discouraged answer for this preregistration form.***

- Yes, at least some data have been collected for this study already
- No, no data have been collected for this study yet
- It's complicated

It's complicated. We have already collected some data but explain in Question 8 why readers may consider this a valid pre-registration nevertheless.

2) ***Hypothesis. What's the main question being asked or hypothesis being tested in this study?***

The main question of this meta-analysis is: What is the association between child maltreatment and loneliness across the lifespan? Based on the literature, we expect that people who have experienced child maltreatment generally feel lonelier than people who have not experienced child maltreatment.

In addition, we will investigate whether the following moderators (for more details about the moderators see Conditions) influence the magnitude of the association between child maltreatment and loneliness:

- Maltreatment variables: Type of maltreatment, perpetrator, age of onset, severity, frequency, and chronicity of the maltreatment.
- Loneliness variables: Type of loneliness and relationship-specific types of loneliness.

- Sample characteristics: Age of the participants when the study was conducted, gender, and socioeconomic, ethnic and clinical background of the participants.
- Study characteristics: Year of publication, country in which the study was conducted, study design, reporter, and reliability of the used measures.

3) ***Dependent variable. Describe the key dependent variable(s) specifying how they will be measured.***

We will calculate standardized mean differences (Cohen's d) for loneliness between maltreated and non-maltreated participants.

Therefore, the following information will be coded:

- Number of participants in the maltreated and non-maltreated group.
- Mean loneliness scores of the maltreated and non-maltreated group.
- Standard deviations of the loneliness scores of the maltreated and non-maltreated group.

If this information is not available, other statistical information which enables us to calculate the standardized mean differences will be coded.

4) ***Conditions. How many and which conditions will participants be assigned to?***

For the moderator analyses, the following information will be coded:

- Type of maltreatment, categorized as 1 = Emotional abuse, 2 = Physical abuse, 3 = Sexual abuse, 4 = Neglect (emotional/physical), 5 = Multitype maltreatment.
- Perpetrator of the maltreatment, categorized as 1 = Parent, 2 = Family member except parents, 3 = Other, 4 = Multiple perpetrators.

- Age of onset of maltreatment, severity, frequency and chronicity of the maltreatment.
- Type of loneliness, categorized as 1 = Emotional loneliness, 2 = Social loneliness, 3 = General loneliness.
- Relationship-specific types of loneliness, categorized as 1 = Peers, 2 = Family, 3 = Romantic partner.
- Gender of the participants. Percentages of males and percentages of females.
- Mean age and standard deviation of age of the participants when the study was conducted. For longitudinal studies the age of the participants at the first wave at which loneliness was measured. Age categories (based on the mean age of the sample), that is, 1 = Children (up to 11.9 years); 2 = Adolescence (12-17.9 years); 3 = Emerging adulthood (18-24.9 years); 4 = Young adulthood (25-44.9 years); 5 = Middle adulthood (45-64.9 years); 6 = Old age (65 or older).
- Socioeconomic status (SES) of the participants, categorized as 1 = > 75% of the participants is of low SES, 2 = > 75% of the participants is of middle/high SES, 3 = Mixed, none of the SES categories include more than 75% of the participants.
- Ethnic background of the participants, categorized as 1 = > 75% of the participants have an ethnic minority background, 2 = > 75% of the participants have an ethnic majority background, 3 = Mixed, no more than 75% of the participants have either an ethnic minority nor an ethnic majority background.
- Clinical background of the participants, categorized as 1 = Participants were not sampled from the general population, 2 = Participants were sampled from the general population. If participants were not sampled from the general population, a more specific code will provided, that is 1 = Maltreated group and control group is included, 2 = Clinical group* (other than a maltreated group) and control group

is included, 3 = Only clinical group (other than a maltreated group) is included, 4 = Other.

* Clinical groups include mental health problems, clinical health problems, and special educational needs.

- Year of publication
- Country in which the study was conducted
- Study design, categorized as 1 = Cross-sectional, 2 = Experimental, 3 = Longitudinal.
- Maltreatment measure, categorized as 1 = Retrospective, 2 = Prospective.
- Reporter of loneliness, categorized as 1 = Self-report, 2 = Parental report, 3 = Teacher report, 4 = Observer, 5 = Peer(s).
- Reporter of the maltreatment, categorized as 1 = Self-report, 2 = Parental report, 3 = Records.
- Reliability of the used measures (Cronbach's alpha).

5) ***Analyses. Specify exactly which analyses you will conduct to examine the main question/hypothesis.***

We will conduct multilevel meta-analyses to account for possible dependency between effect sizes because most studies report multiple effect sizes. Specifically, we will use a three-level model including sampling variance (Level 1), within-study variance (Level 2), and between-study variance (Level 3). The analyses will be conducted with the metafor package in R. Moderating effects will be tested one by one.

Regarding the categorical moderators, when fewer than five effect sizes are available for a particular category, subsequent categories will be merged for ordinal moderators, or small categories will be excluded from the analysis for nominal moderators.

6) ***Outliers and Exclusions. Describe exactly how outliers will be defined and handled, and your precise rule(s) for excluding observations.***

We will examine whether there are outliers, that is, effect size scores deviating three SDs or more from the mean. When there are outliers in the dataset, we will conduct the main analyses on all available data, and on a dataset without the outliers, and compare and report the results of both analyses. The moderator analyses will also be conducted on the data with and without outliers. If the conclusions of the moderator analyses are impacted by the outlying values, we will report results of the moderator analyses without outliers.

7) ***Sample Size. How many observations will be collected or what will determine sample size? No need to justify decision, but be precise about exactly how the number will be determined.***

The size of the dataset is determined by how many articles of the literature search are eligible for this meta-analysis. The final size of the dataset will depend on how many of the eligible articles provide enough statistical information to calculate an effect size (standardized mean differences). We will utilize the PRISMA guidelines for a systematic review.

8) ***Other. Anything else you would like to pre-register? (e.g., secondary analyses, variables collected for exploratory purposes, unusual analyses planned?)***

The literature search for the present meta-analysis and the selection of eligible articles have been conducted. Coding of articles had only recently started.

Searches will be conducted in eight databases (PsycINFO, ERIC, PubMed, Web of Science, MEDLINE, EMBASE, Cochrane Central Register of Controlled Trials). The keywords include loneliness variables (lonel* or "perceived social isolation"), maltreatment variables (maltreat* or mistreat* or abus* or neglect* or abandoned or incest* or rape* or "shaken baby syndrome"), and child/adolescent variables (child* or kid* or teen* or adolescen* or youth). The search is limited to title, abstract, and keywords.

Inclusion criteria for the first selection (title and abstract) are:

1. Study is published in English
2. Study is published in a peer-reviewed journal
3. Study is quantitative research

Inclusion criteria for the second selection (full text) are:

1. Measurement of loneliness must be present
2. Measurement of child maltreatment (emotional abuse, physical abuse, sexual abuse and/or neglect) must be present
3. Both maltreated and non-maltreated participants must be included in the study

9) ***Name. Give a title for this AsPredicted pre-registration***

The association between child maltreatment and loneliness across the lifespan: A multilevel meta-analysis

Finally. For record keeping purposes, please tell us the type of study you are pre-registering.

- Class project or assignment
- Experiment
- Survey
- Observational/archival study
- Other

Other. If 'other' was selected above, describe the type of study you're registering.

Meta-analysis
