# Supplementary material for: The Association Between Child Maltreatment and Loneliness Across the Lifespan: A Systematic Review and Multilevel Meta-Analysis
Source: Child Maltreat. 2022 Jun 2;29(2):388–404. doi: 10.1177/10775595221103420 (PMC11539460; doi:10.1177/10775595221103420)
Supplement: sj-pdf-2-cmx-10.1177_10775595221103420 – Supplemental Material for The Association Between Child Maltreatment and Loneliness Across the Lifespan: A Systematic Review and Multilevel Meta-Analysis [file sj-pdf-2-cmx-10.1177_10775595221103420.pdf]

Appendix A. Database Search Strings

PsycINFO / ERIC / MEDLINE

((lonel* or "perceived social isolation") and (maltreat* or mistreat* or abus* or neglect* or abandoned or incest* or rape* or "shaken baby syndrome") and (child* or kid* or teen* or adolescen* or youth)).mp.

Cochrane Library

Searched in ‘title abstract keyword’ ((lonel* or "perceived social isolation") and (maltreat* or mistreat* or abus* or neglect* or abandoned or incest* or rape* or "shaken baby syndrome") and (child* or kid* or teen* or adolescen* or youth)).

EMBASE

(lonel*:ti,ab,kw,lnk OR 'perceived social isolation':ti,ab,kw,lnk) AND (maltreat*:ti,ab,kw,lnk OR mistreat*:ti,ab,kw,lnk OR abus*:ti,ab,kw,lnk OR neglect*:ti,ab,kw,lnk OR abandoned:ti,ab,kw,lnk OR incest*:ti,ab,kw,lnk OR rape*:ti,ab,kw,lnk OR 'shaken baby syndrome':ti,ab,kw,lnk) AND (child*:ti,ab,kw,lnk OR kid*:ti,ab,kw,lnk OR teen*:ti,ab,kw,lnk OR adolescen*:ti,ab,kw,lnk OR youth:ti,ab,kw,lnk).

PubMed

((lonel* [TIAB] or "perceived social isolation" [TIAB]) and (maltreat* [TIAB] or mistreat* [TIAB] or abus* [TIAB] or neglect* [TIAB] or abandoned [TIAB] or incest* [TIAB] or rape* [TIAB] or "shaken baby syndrome" [TIAB]) and (child* [TIAB] or kid [TIAB] or teen* [TIAB] or adolescen* [TIAB] or youth [TIAB])) OR ((lonel* [SH] or "perceived social isolation" [SH]) and (maltreat* [SH] or mistreat* [SH] or abus* [SH] or neglect* [SH] or abandoned [SH] or incest* [SH] or rape* [SH] or "shaken baby syndrome" [SH]) and (child* [SH] or kid [SH] or teen* [SH] or adolescen* [SH] or youth [SH])).

Web of Science

TS = ((lonel* or "perceived social isolation") and (maltreat* or mistreat* or abus* or neglect* or abandoned or incest* or rape* or "shaken baby syndrome") and (child* or kid* or teen* or adolescen* or youth)).

Appendix B. Concise Version of the Coding Manual

Child Maltreatment Characteristics

Type of child maltreatment	1 = Emotional abuse<br>2 = Physical abuse<br>3 = Sexual abuse<br>4 = Emotional neglect<br>5 = Physical neglect<br>6 = Multitype maltreatment (i.e., multiple types of child maltreatment were combined)
Perpetrator	1 = Parent<br>2 = Family member except parents<br>3 = Other<br>4 = Multiple perpetrators
Age of onset	Note all available information
Severity	Note all available information
Frequency	Note all available information
Chronicity	Note all available information

Loneliness Characteristics

Type of loneliness	1 = Emotional loneliness<br>2 = Social loneliness<br>3 = General loneliness (i.e., emotional and social loneliness have been combined into one score)

Relationship-specific type of loneliness	1 = Peers<br>2 = Family<br>3 = Romantic partner

Study Characteristics

Measure child maltreatment	1 = Retrospective (i.e., participants indicated maltreatment in their past)<br>2 = Prospective (i.e., the child maltreatment was recorded at the time the maltreatment occurred, e.g., via Child Protective Services records or participants indicated that they currently experience maltreatment)
Reporter loneliness	1 = Self-report<br>2 = Parental report<br>3 = Teacher report<br>4 = Observer<br>5 = Peer(s)
Reporter child maltreatment	1 = Self-report<br>2 = Parental report<br>3 = Records (e.g., Child Protective Services, court)
Study design	1 = Cross-sectional<br>2 = Experimental<br>3 = Longitudinal<br>Also studies that used data on maltreatment that is collected in the past (e.g., Child Protective Services records) and measured loneliness at the time of the study will be considered as longitudinal.
Reliability measure of loneliness	Cronbach's alpha

Reliability measure of child maltreatment	Cronbach's alpha
Country	
Year of publication	
**Sample Characteristics**	
Sample size	Number of participants
Gender	Percentages of females
Age	Mean<br>Standard deviation
Socioeconomic status (SES)	1 = 75% or more of the participants had low SES<br>2 = 75% or more of the participants had middle/high SES<br>3 = Mixed, none of the SES categories included more than 75% of the participants
Ethnic background	1 = 75% or more the participants had an ethnic minority background<br>2 = 75% or more of the participants had an ethnic majority background<br>3 = Mixed, neither participants with an ethnic majority nor ethnic minority background made up more than 75% of the sample
Clinical status	1 = Nonclinical

2 = Clinical

3 = Mixed, both clinical and nonclinical samples were included

Appendix C. Study and Sample Characteristics for each Included Study

Article	Country	*N*	Proportion female	Age mean	Age range	SES	Ethnic background	Clinical status	Study design	CM measure	CM reporter	CM type	CM alpha	LO type	LO alpha	*g*	*SE*
Aakvaag et al. (2018)	NO	1,011	.60	21.00	17-35	NA	NA	NC	L	R	S	SA	NA	S	.85	0.64	0.08
												PA	NA	S	.85	0.85	0.16
												EA	NA	S	.85	0.64	0.09
												EN	NA	S	.85	0.77	0.11
Appleyard et al. (2010)	US	1,354	.51	6.00	NA	NA	NA	NC	L	P	R	EA, PA, SA, N	NA	S	NA	0.24	0.06
Babad et al. (2020)	US	436	.64	19.73	18-25	M	M	NC	C	R	S	EA	NA	NA	.85	0.59	0.11
												PA	NA	NA	.85	0.25	0.16
												SA	NA	NA	.85	0.27	0.14
												EN	NA	NA	.85	0.84	0.14
												PN	NA	NA	.85	0.52	0.16
Bell & Belicki (1998)	CA	109	.71	45.00	14-78	M	NA	NC	C	R	S	EA, PA, SA	NA	G	NA	0.54	0.21
Blanchard-Dallaire et al. (2014)	CA	168	.70	9.79	8-12	M	NA	NC	L	P	R	SA	NA	S	.89	0.20	0.15
Boyda et al. (2015)	UK	7,403	.57	51.00	16+	MH	MA	NC	C	R	S	SA	NA	S	NA	0.18	0.15
Brown et al. (2016)	US	339	.49	19.00	18-25	NA	M	NC	C	R	S	PA	.81	G	.96	0.52	0.12
												PN	.59	G	.96	0.26	0.11
												SA	.94	G	.96	0.26	0.11
												EA	.86	G	.96	0.60	0.12
												EN	.85	G	.96	0.58	0.12
Chen & Qin (2020)	CN	569	.49	11.68	10-15	M	NA	NC	C	R	S	EA	.75	S	.82	0.61	0.09
Fontes et al. (2017)	BR	2,575,269	.52	NA	NA	NA	NA	NC	C	R	S	SA	NA	G	NA	0.59	0.00
Garnefski & Arends (1998)	NL	1,490	.80	15.00	12-19	M	NA	M	C	R	S	SA	NA	G	.60	0.52	0.05

Article	Country	*N*	Proportion female	Age mean	Age range	SES	Ethnic background	Clinical status	Study design	CM measure	CM reporter	CM type	CM alpha	LO type	LO alpha	*g*	*SE*
Giano & Hubach (2019)	US	156	.00	35.38	18+	M	MA	NC	C	R	S	EA	NA	G	.94	0.54	0.16
												PA	NA	G	.94	0.22	0.18
												SA	NA	G	.94	0.05	0.18
Gibson & Hartshorne (1996)	US	257	1.00	NA	NA	MH	MA	NC	C	R	S	SA	NA	G	.94	0.90	0.16
Grayston et al. (1992)	CA	70	1.00	NA	7-12	L	NA	NC	L	P	R	SA	NA	S	NA	0.11	0.24
Hamdan-Mansour & Marmash (2007)	JO	645	.49	NA	NA	NA	NA	NC	C	R	S	SA	NA	NA	NA	-0.09	0.22
												SA	NA	NA	NA	-0.01	0.25
												SA	NA	NA	NA	-0.11	0.23
Hanlon et al. (2020)	UK	179,801	.58	56.80	AN	M	NA	NC	C	R	S	PA	NA	G	NA	0.25	0.03
												EA	NA	G	NA	0.38	0.03
												SA	NA	G	NA	0.18	0.04
												N	NA	G	NA	0.35	0.04
Hart et al. (2018)	CA	304	.00	44.15	NA	M	MA	NC	C	R	S	EA	.91	G	.96	0.45	0.12
												PA	.91	G	.96	0.52	0.12
												SA	.91	G	.96	0.20	0.12
Holmberg & Hellberg (2010)	SE	1,428	1.00	15.63	13-18	M	NA	NC	C	R	S	SA	NA	G	NA	0.38	0.02
Howe & Parke (2001)	US	78	.42	8.70	4.3-11.6	L	M	NC	C	R	S	EA, PA, SA, N	NA	S	.90	1.22	0.25
Jacob et al. (2020)	UK	7,403	.51	46.30	16+	NA	MA	NC	C	R	S	SA, PA	NA	S	NA	0.52	0.00
Kamiya et al. (2016)	IE	6,904	.54	63.95	50+	M	NA	NC	C	R	S	SA	.79	G	NA	1.68	0.05
Khan et al. (2020)	BD	2,989	.35	NA	11-18	NA	NA	NC	C	R	S	PA	NA	G	NA	0.42	0.01
Kircaburun et al. (2019)	TR	252	.07	18.87	13-38	NA	NA	NC	C	R	S	EA, EN	.87	G	.68	1.03	0.16

Article	Country	*N*	Proportion female	Age mean	Age range	SES	Ethnic background	Clinical status	Study design	CM measure	CM reporter	CM type	CM alpha	LO type	LO alpha	*g*	*SE*
Kivelä et al. (2019)	NL	2,130	.67	42.40	18-65	NA	MA	M	L	R	S	EA	NA	G	NA	0.33	0.14
												PA	NA	G	NA	0.40	0.14
												SA	NA	G	NA	0.30	0.13
												EN	NA	G	NA	0.54	0.14
												PN	NA	G	NA	0.43	0.14
Leve et al. (2007)	US	121	.49	5.90	NA	NA	MA	NC	C	P	R	EA, PA, SA, N	NA	S	.51	0.05	0.20
												EA, PA, SA, N	NA	S	NA	0.38	0.19
Lin et al. (2020)	TW	2,289	.52	31.32	30-35	MH	NA	NC	L	R	S	EN	NA	E	.51	0.07	0.05
												EN	NA	S	.77	0.23	0.05
												EA	NA	E	.51	0.22	0.08
												EA	NA	S	.77	0.18	0.08
												PA	NA	E	.51	0.11	0.06
												PA	NA	S	.77	0.14	0.06
Lin et al. (2016)	CN	256	.28	9.56	NA	M	NA	C	C	R	P	EN	.81	S	.91	0.61	0.14
												EA	.84	S	.91	0.86	0.15
												PA	.92	S	.91	0.68	0.14
Loos & Alexander (1997)	US	402	.61	19.26	16-56	MH	M	NC	C	R	S	PA	NA	G	.94	0.50	0.11
												EN	NA	G	.94	0.88	0.12
Løyland (2016)	NO	369	.41	33.87	18-60	L	MA	NC	C	R	S	SA	NA	G	NA	0.33	0.15
												EA, PA	NA	G	NA	0.47	0.13
Luo et al. (2020)	CN	1,302	.50	11.31	9-13	NA	NA	NC	C	R	S	EA	.84	S	.88	0.58	0.06
Ma et al. (2020)	CN	981	.49	13.68	NA	NA	NA	NC	C	R	S	EA, PA, SA, N	.79	S	.75	1.22	0.09
Malta et al. (2019)	BR	102,301	.51	NA	NA	NA	NA	NC	C	R	S	PA	NA	G	NA	4.11	0.01
Malta et al. (2014)	BR	109,104	.52	NA	NA	NA	NA	NC	C	R	S	PA	NA	G	NA	0.46	0.00

Article	Country	*N*	Proportion female	Age mean	Age range	SES	Ethnic background	Clinical status	Study design	CM measure	CM reporter	CM type	CM alpha	LO type	LO alpha	*g*	*SE*
Mangueira & Lopes (2016)	BR	110	.05	43.98	19-65	L	NA	M	C	R	S	SA	NA	G	NA	-0.51	0.35
												PA	NA	G	NA	-0.42	0.09
Matthews et al. (2020)	UK	2,232	.51	12.00	NA	M	MA	NC	L	P	NA	PN	NA	S	NA	0.28	0.07
												PA	NA	S	NA	0.18	0.05
												SA	NA	S	NA	0.19	0.18
Patanwala et al. (2018)	US	283	.24	59.00	50+	L	MI	NC	L	R	S	PA	NA	NA	NA	0.12	0.13
												EA	NA	NA	NA	0.25	0.12
												SA	NA	NA	NA	0.21	0.18
Rafi et al. (2017)	IR	492	.63	13.60	NA	NA	NA	NC	C	R	S	EA	.96	NA	.66	1.32	0.13
Rew (2002)	US	96	.47	17.80	12-23	NA	M	NC	C	R	S	SA	NA	G	.86	0.45	0.22
Rogers et al. (2007)	UK	253	.39	38.40	17-82	NA	MA	NC	C	R	S	EA, PA, SA, N	.95	S	.91	0.72	0.14
												EA, PA, SA, N	.95	E	.89	1.06	0.16
												EA, PA, SA, N	.95	E	.89	0.63	0.14
Schaan et al. (2019)	LU / DE	121	1.00	23.00	NA	NA	NA	NC	C	R	S	EA	.89	G	.88	0.53	0.20
												PA	.63	G	.88	0.64	0.25
												SA	.92	G	.88	0.39	0.30
												EN	.86	G	.88	0.92	0.24
												PN	.30	G	.88	0.87	0.19
Scheer & Antebi-Gruszka (2019)	US	207	.13	27.60	18-78	M	M	NC	C	R	S	PA	NA	G	.76	0.31	0.12
Schuck & Widom (2001)	US	582	1.00	29.11	18-40	M	M	NC	L	P	R	PA, SA, N	NA	S	NA	0.10	0.08

Article	Country	*N*	Proportion female	Age mean	Age range	SES	Ethnic background	Clinical status	Study design	CM measure	CM reporter	CM type	CM alpha	LO type	LO alpha	*g*	*SE*
Steenkamp et al. (2019)	NL	59	.42	31.80	19-57	NA	NA	C	C	R	S	PA	NA	G	NA	0.90	0.30
												SA	NA	G	NA	0.15	0.38
												EA	NA	G	NA	0.17	0.28
Stensland et al. (2014) (Data available from the HUNT study)	NO	10,464	.50	15.80	12-20	MH	MA	NC	C	R	S	SA	NA	G	NA	0.73	0.06
												SA	NA	G	NA	0.80	0.07
												PA	NA	G	NA	0.55	0.04
Stults et a. (2015)	US	528	.00	NA	18-19	M	M	NC	C	R	S	EA, PA, SA, N	NA	G	.67	0.41	0.09
Tang et al. (2018)	CN	15,623	.49	15.10	12-18	MH	MA	NC	C	R	S	M, N	.81	NA	.76	0.26	0.02
												N	.81	NA	.76	0.38	0.02
Tidefors et al. (2011)	SE	45	.00	16.20	13-22	NA	M	C	L	P	R	EA, N	NA	E	NA	-0.10	0.34
												PA	NA	E	NA	0.09	0.29
												SA	NA	E	NA	-0.34	0.30
Træen et al. (2020) - Study 1	NO	1,271	.48	66.80	60-75	NA	NA	NC	C	R	S	SA	NA	S	.80	0.28	0.10
Træen et al. (2020) - Study 2	DK	1,045	.48	67.80	60-75	NA	NA	NC	C	R	S	SA	NA	S	.80	0.39	0.12
Træen et al. (2020) - Study 3	BE	991	.48	66.85	60-75	NA	NA	NC	C	R	S	SA	NA	S	.80	0.83	0.13
Træen et al. (2020) - Study 4	PT	509	.47	66.71	60-75	NA	NA	NC	C	R	S	SA	NA	S	.80	0.06	0.27
Van der Veen et al. (2015)	NL	350	.66	70.70	60+	NA	NA	C	C	R	S	EN	NA	G	NA	0.25	0.11
												PA	NA	G	NA	0.10	0.14
												EA	NA	G	NA	0.15	0.12
												SA	NA	G	NA	0.12	0.13

Article	Country	*N*	Proportion female	Age mean	Age range	SES	Ethnic background	Clinical status	Study design	CM measure	CM reporter	CM type	CM alpha	LO type	LO alpha	*g*	*SE*
Wong et al. (2019)	US	308	.46	35.49	18-70	M	MA	NC	C	R	S	EN	NA	G	.92	0.82	0.13
												PN	NA	G	.92	0.70	0.20
												EA	NA	G	.92	0.88	0.13
												PA	NA	G	.92	0.42	0.14
												SA	NA	G	.92	0.38	0.18
Zhang et al. (2020)	CN	1,019	.47	18.60	18-21	M	NA	NC	C	R	S	EN	NA	G	.84	0.39	0.12
												PN	NA	G	.84	-0.19	0.16
												EA	NA	G	.84	0.73	0.14
												PA	NA	G	.84	0.46	0.07
												SA	NA	G	.84	0.37	0.10

Note. CM = child maltreatment. LO = loneliness. NA = not available. SES (socioeconomic status): L = >75% low; M = mixed; MH = >75% middle/high. Ethnic background: M = mixed; MA = >75% majority; MI = >75% minority. Clinical status: C = clinical; M = mixed; NC = nonclinical. Study design: C = cross-sectional; L = longitudinal. Child maltreatment measure: P = prospective; R = retrospective. Child maltreatment reporter: P = parental report; R = records; S = self-report. Child maltreatment type: EA = emotional abuse; EN = emotional neglect; M = maltreatment (not further specified); N = neglect (not further specified); PA = physical abuse; PN = physical neglect; SA = sexual abuse. Loneliness type: E = emotional; G = general; S = social.
